# Million Veteran Program’s response to COVID-19: Survey development and preliminary findings

**DOI:** 10.1371/journal.pone.0266381

**Published:** 2022-04-25

**Authors:** Stacey B. Whitbourne, Xuan-Mai T. Nguyen, Rebecca J. Song, Emily Lord, Michelle Lyden, Kelly M. Harrington, Rachel Ward, Yanping Li, Jessica V. V. Brewer, Kelly M. Cho, Luc Djousse, Sumitra Muralidhar, Philip S. Tsao, J. Michael Gaziano, Juan P. Casas, on behalf of the MVP COVID-19 Science Program

**Affiliations:** 1 Massachusetts Veterans Epidemiology Research and Information Center (MAVERIC), VA Boston Healthcare System, Boston, MA, United States of America; 2 Department of Medicine, Harvard Medical School, Boston, MA, United States of America; 3 Division of Aging, Department of Medicine, Brigham and Women’s Hospital, Boston, MA, United States of America; 4 Carle Illinois College of Medicine, University of Illinois, Champaign, IL, United States of America; 5 Department of Epidemiology, Boston University School of Public Health, Boston, MA, United States of America; 6 Department of Psychiatry, Boston University School of Medicine, Boston, MA, United States of America; 7 New England Geriatric Research, Education, and Clinical Center (GRECC), VA Boston Healthcare System, Boston, MA, United States of America; 8 Department of Physical Medicine and Rehabilitation, Harvard Medical School, Boston, MA, United States of America; 9 Office of Research and Development, Veterans Health Administration, Washington, D.C., United States of America; 10 VA Palo Alto Epidemiology Research and Information Center for Genomics, VA Palo Alto Health Care System, Palo Alto, CA, United States of America; 11 Department of Medicine, Stanford University School of Medicine, Stanford, CA, United States of America; 12 Stanford Cardiovascular Institute, Stanford University School of Medicine, Stanford, CA, United States of America; Universite du Quebec a Montreal, CANADA

## Abstract

**Background:**

In response to the novel Coronavirus Disease 2019 (COVID-19) pandemic, the Department of Veterans Affairs (VA) Million Veteran Program (MVP) organized efforts to better understand the impact of COVID-19 on Veterans by developing and deploying a self-reported survey.

**Methods:**

The MVP COVID-19 Survey was developed to collect COVID-19 specific elements including symptoms, diagnosis, hospitalization, behavioral and psychosocial factors and to augment existing MVP data with longitudinal collection of key domains in physical and mental health. Due to the rapidly evolving nature of the pandemic, a multipronged strategy was implemented to widely disseminate the COVID-19 Survey and capture data using both the online platform and mailings.

**Results:**

We limited the findings of this paper to the initial phase of survey dissemination which began in May 2020. A total of 729,625 eligible MVP Veterans were invited to complete version 1 of the COVID-19 Survey. As of October 31, 2020, 58,159 surveys have been returned. The mean and standard deviation (SD) age of responders was 71 (11) years, 8.6% were female, 8.2% were Black, 5.6% were Hispanic, and 446 (0.8%) self-reported a COVID-19 diagnosis. Over 90% of responders reported wearing masks, practicing social distancing, and frequent hand washing.

**Conclusion:**

The MVP COVID-19 Survey provides a systematic collection of data regarding COVID-19 behaviors among Veterans and represents one of the first large-scale, national surveillance efforts of COVID-19 in the Veteran population. Continued work will examine the overall response to the survey with comparison to available VA health record data.

## Introduction

The Coronavirus Disease 2019 (COVID-19) pandemic caused by Severe Acute Respiratory Syndrome Coronavirus 2 (SARS-CoV-2) [1–3] has had an immense impact on individuals and healthcare systems globally. As of February 2021, more than 2 million deaths related to COVID-19 were reported worldwide. The rapidly evolving pandemic has affected daily life with the implementation of COVID-19-specific public health practices [4, 5]. It has also presented many challenges in adapting medical care and treatment for different populations. In response to the pandemic in early 2020, the Department of Veterans Affairs (VA) Million Veteran Program (MVP) began efforts to understand how individual and societal reactions to the novel virus could impact both the short- and long-term health and well-being of US Veterans. The goal was to capture information not available in VA electronic health records (EHR) to complement existing EHR COVID-19 related data.

While previous work has investigated the effect of the COVID-19 pandemic on changing health behaviors and lifestyle factors through survey data [6–12], our research is the first large-scale, national survey of COVID-19 in a US Veteran population. The VA is the largest single payer health system within the US. The VA EHR contains over 20 years of data on 6 million annual active users nationally. Within MVP, we have previously used EHR data to identify several risk factors for COVID-19 severity and progression [13]. The addition of this survey data will facilitate investigation of how various COVID-19-related health behaviors, lifestyle changes, and psychosocial factors impact short- and long-term health outcomes.

The VA MVP is an ongoing national voluntary research program that aims to better understand how genetic, lifestyle, and environmental factors influence Veteran health.

The framework and methods used for MVP recruitment and enrollment, as described elsewhere [14, 15], allow the program to serve as a rapid response platform for pandemic data collection. Current MVP data collection efforts span across multiple modalities including postal mail, email, and secure online web portals. Veterans enrolled in MVP consent to providing (1) a blood sample for biobanking, (2) responses to surveys, (3) access to data from health record databases (VA health records to start, with the potential for access to additional health record databases in the future), and (4) permission to be re-contacted for further data collection or participation in additional MVP-related research. MVP is embedded within the Veterans Health Administration (VHA), the largest integrated national health system in the country, with access to national VA clinical and administrative databases. In addition to being a research program, MVP provides scientific, regulatory, technological, and administrative infrastructure for longitudinal data collection (including medical history, physical examination, laboratory, diagnostic information) among a growing research community. This infrastructure is uniquely equipped to systematically handle ingestion of self-reported data not available in health records along with clinical health record data. Given the platform’s robustness, it can easily integrate unanticipated data collection such as a COVID-19 survey with ongoing research efforts. The goal of this paper is to describe the development and implementation of version 1 of the MVP COVID-19 Survey and to present preliminary findings from the first six months of survey data collection.

## Methods

### Survey planning

Development of the initial version of the MVP COVID-19 Survey began with a review of resources pertaining to COVID-19 including recommendations from the World Health Organization (WHO), Centers for Disease Control and Prevention (CDC), U.S. Food and Drug Administration (FDA), U.S. National Surveillance and Reporting Agency, and the CDC National Notifiable Diseases Surveillance System. A panel of VA and non-VA experts in the areas of infectious diseases, pulmonary functioning, cardiology, nephrology, mental health, and epidemiology was consulted to refine domains of interest for the survey.

### Survey design and measures

The MVP COVID-19 Survey includes elements from the MVP Baseline and Lifestyle Surveys along with additional measures to assess COVID-19 clinical and psychosocial outcomes. Version 1 of the survey is described in this work. Consisting of 54-items, the survey is divided into the following sections: 1) demographic information, lifestyle behaviors, and military status; 2) COVID-19 exposures and household contacts; 3) COVID-19 symptoms and diagnosis; 4) COVID-19 hospitalization and medical interventions; 5) social distancing behaviors and psychosocial well-being; and 6) medical conditions, healthcare utilization, health insurance, and subjective health status. Table 1 describes the domains of the MVP COVID-19 Survey and their respective sources. The survey sections and subsequent questions were designed to flow in sequential order of relevance using skip pattern logic and conditional and unconditional branching methods. Survey questions were formatted to include close-ended, multiple choice, and brief fill‐ins. Question response options vary based on the nature of the questions and appropriate level of measurement needed to create the survey, including dichotomous nominal, fixed ordinal and rank ordered response scales. Diagnosis of COVID-19 was adapted from the COronavirus Pandemic Epidemiology (COPE) Consortium Tool [16] and defined as answering “Yes, confirmed by a positive laboratory test” or “Yes, suspected by a doctor but not confirmed by a test” to question 29 of the survey which asks “Have you been diagnosed with COVID-19?”. The complete MVP COVID-19 Survey is available in S1 Appendix.

**Table 1:** Summary of MVP COVID-19 Survey items and sources.

Domain	Measure	Source
Personal Information	Sociodemographic	MVP Baseline Survey [14, 15]; National Health Interview Survey [19].
	Branch of the military Deployment/activation	
	Alcohol use	
	Tobacco use	
	E-cigarette/vaping	
COVID-19 Infected	Exposure	Adapted from the COronavirus Pandemic Epidemiology (COPE) Consortium Tool [16].
	Symptoms	
	Diagnosis	
	Medical treatment	
COVID-19 Impact Well-being	Depression/anxiety	Patient Health Questionnaire (PHQ-4) [21]; PROMIS^®^ Measures v2.0 Social Isolation and Emotional Support [20]; Loss of Resource Scale [22].
	Social and emotional support	
	Health-related quality of life	
	Loss of resources	
COVID-19 Related Behaviors	Social distancing/self-protective behaviors	Adapted from CDC COVID-19 Community Survey Question Bank [17]; Epidemic Pandemic Impacts Inventory (EPII) [18].
	Daily life changes	
Medical Conditions and Comorbidity	Health status	MVP Baseline Survey [14, 15].
	Medical history	
Healthcare Usage	Healthcare usage	MVP Baseline Survey [14, 15].
	Health insurance	

A variety of validated instruments were included to measure the psychosocial impact of the COVID-19 pandemic: 1) social distancing practices adapted from the CDC COVID-19 Community Survey Question Bank [17]; 2) daily life changes from the Epidemic Pandemic Impacts Inventory [18]; 3) alcohol and tobacco use questions from the National Health Interview Survey [19]; (4) social isolation and emotional support items from the PROMIS measures [20]; (5) depression and anxiety symptoms from the PHQ-4 [21]; and (6) loss of tangible (e.g., property, finances) and non-tangible (e.g., family stability, community engagement) resources experienced adapted from the Loss of Resource Scale [22]. The last section of the survey is adapted from the MVP Baseline Survey and includes questions related to medical conditions, healthcare utilization, and subjective health status. Additions to the medical conditions included items related to Gulf War Illness, chronic fatigue syndrome, fibromyalgia and vaccines received during military service.

### Sample population

**Fig 1:**
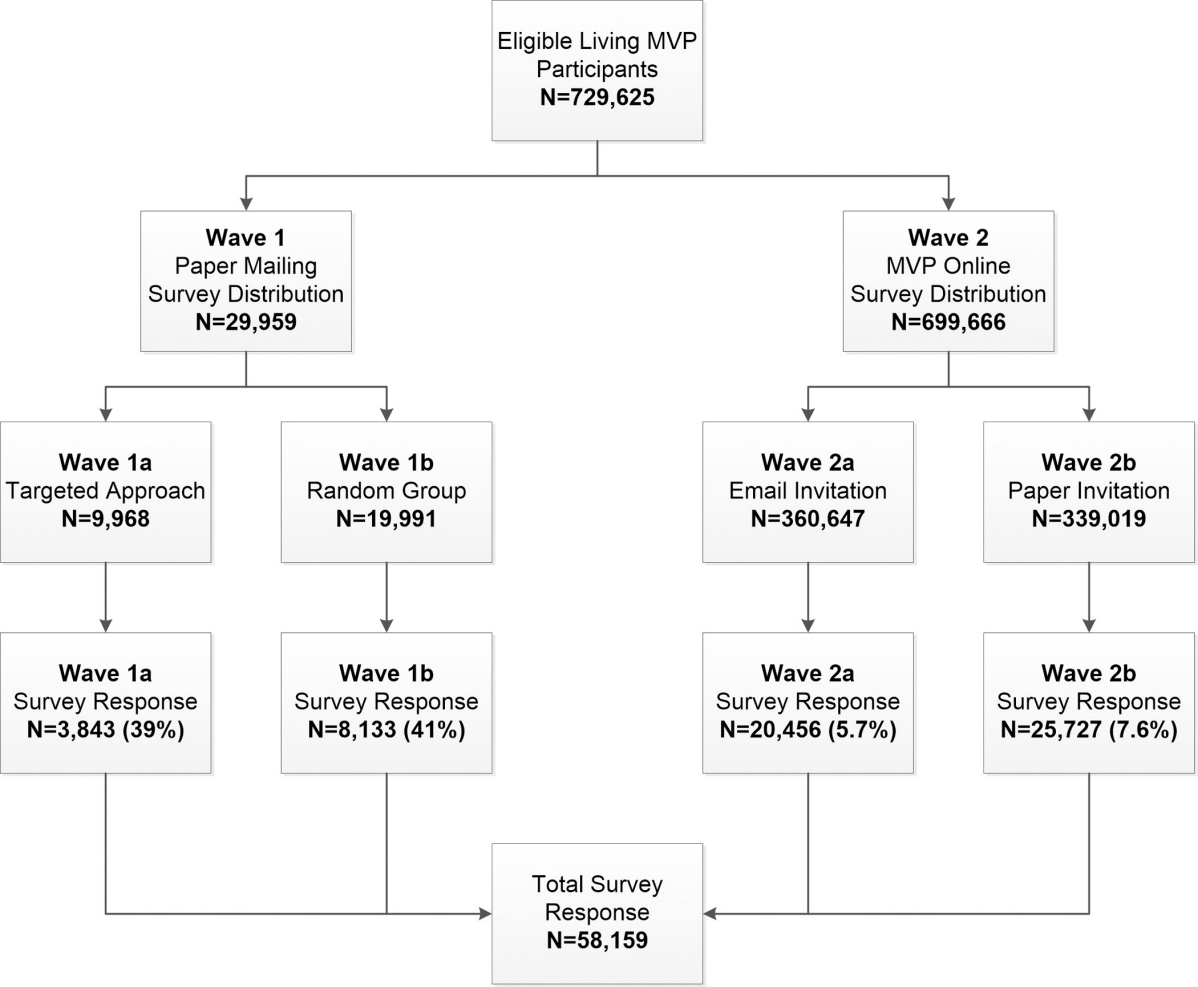
MVP COVID-19 Survey distribution flow.

Detailed descriptions of the MVP cohort have been published previously [14, 15]. MVP is approved through the VA Central Institutional Review Board. All participants provide written informed consent/HIPAA authorization at the time of enrollment which constitutes: 1) provision of a blood specimen for analysis; 2) collection of self-report data through MVP Surveys; 3) access to health records; and 4) ability for future contact for additional research purposes. All eligible, living, and non-withdrawn MVP participants received invitations to complete the MVP COVID-19 Survey (n = 729,625) in May through June of 2020. Fig 1 demonstrates the flow for contact of the first version of the survey. Wave 1 of the survey distribution (n = 29,959), launched May 5, 2020, implemented a targeted approach to contacting Veterans identified as COVID-19 persons of interest. The selection included Veterans (Wave 1a; n = 9,968) who either tested for COVID-19 at a VA facility or resided in states considered to be a COVID-19 “hot spot” at the time including New York, Connecticut, Maine, Massachusetts, New Hampshire, Vermont, Rhode Island, Illinois, Louisiana, California, and Washington. Information on COVID-19 testing status was obtained from the VA Corporate Data Warehouse (CDW) [23]. The remaining surveys from Wave 1 were sent to a random sample (Wave 1b; n = 19,991) of MVP participants as a comparison group. The remaining eligible MVP participants (n = 699,666) received the survey invitation as part of Wave 2, which launched May 18, 2020.

### Survey deployment

Due to the rapidly evolving nature of the pandemic, a multipronged sampling strategy was employed to quickly capture data on COVID-19 using mailing and online efforts. Paper and online survey modalities were implemented to allow for ease-of-use and completion by participants. The paper survey was released May 5, 2020 and the online version went live May 18, 2020. During Wave 1 of the survey deployment, participants were mailed a paper version of the survey. Wave 2 of the survey deployment adopted a hybrid approach of inviting participants through an email invitation with a link to complete the MVP COVID-19 Survey online (Wave 2a; n = 360,647) or sending a paper invitation asking them to go online to complete the survey (Wave 2b; n = 339,019). All participants invited as part of Wave 2 were provided with the option of requesting a mailed paper survey. Additional rounds of survey invitations have been distributed to MVP participants and will be reported on in future publications. Data presented in this paper are from version 1 of the MVP COVID-19 Surveys collected during a 6-month time frame (May 5, 2020 through October 31, 2020).

### Data analysis

Survey response and demographic data are presented as frequencies and percentages with total responders as the denominator. Public health behaviors related to COVID-19 by self-reported COVID positivity, age, and gender were analyzed from question 41 of the MVP COVID-19 Survey. Group comparisons were calculated by a multivariate adjusted logistic regression model that mutually adjusted for gender (male, female, missing), age (years: <55, 55–64, 65–74, 75+, missing) and self-reported COVID positive status (no, yes, missing) (statistical significance set at *P*<0.05). Likelihood of public health behaviors comparing different groups were presented using odds ratios (OR) and 95% confidence intervals (CI). Missing data were not included in the analyses. All analyses were completed using SAS 9.4 (Cary, North Carolina).

## Results

As of October 31, 2020, a total of 58,159 MVP COVID-19 Surveys were received from the initial round of survey distribution for an overall response rate of 8%. Within the two waves, response rates varied. Among those in Wave 1 who received a paper survey in the mail, 3,843 out of 9,968 (39%) of those identified as COVID-19 persons of interest (Wave 1a) returned surveys. Among Wave 1b (randomly selected group), 8,133 out of 19,991 (41%) returned surveys. We observed that those in the randomly selected group were slightly more likely to respond than the targeted persons of interest (p = 0.0004). In the second wave of survey distribution, where Veterans were invited either by email or paper to take the survey online, 20,456 out of the 360,647 (5.7%) MVP Veterans contacted by email (Wave 2a) completed the survey and 25,727 out of the 339,019 (7.6%) contacted through mail to take the survey online (Wave 2b) completed the survey. We also observed that MVP Veterans contacted directly via email were more likely to complete the survey than those who received the invite to go online by mail (p<0.001). Within the second wave responders, a total of 45,260 (6.5%) Veterans requested a paper version of the survey with 18,529 (40.9%) being returned. Among all survey responders (n = 58,159), 48% were completed online and the remaining surveys were completed by paper.

The demographic profile of MVP COVID-19 Survey responders is presented in Table 2, both overall (n = 58,159) and those who self-reported as having COVID-19 (n = 446). Overall, MVP COVID-19 survey responders were predominantly male (90%), similar to the COVID-19 positive self-reporters (91%). The mean age of all responders was 70.8 (SD: 11.0) years while the self-reported COVID-19 positive responders were younger (67.5; SD: 12.4). The majority of survey responders were White (88%) and non-Hispanic (94%). Out of those who self-reported having COVID-19, the proportion was higher among Black responders compared to White (2.1% vs. 0.6%, p<0.001) and Hispanic vs. Non-Hispanic responders (1.3% vs. 0.7%, p<0.001). A total of 24% of survey responders did not answer question 29 regarding COVID-19 diagnosis.

**Table 2:** Demographic characteristics of MVP COVID-19 Survey responders and self-reported COVID-19 positive.

Characteristics	All Responders	Self-reported COVID-19 diagnosis
	N = 58,169	N = 446
Age, M ± SD	70.8 ± 11.0	67.5 ± 12.4
Age category, n (%)		
<55	5,063 (9)	61 (14)
55–64	8,260 (14)	106 (24)
65–74	25,282 (44)	163 (37)
75+	18,625 (33)	109 (25)
Gender, n (%)		
Male	52,737 (91.4)	402 (91)
Female	4,865 (8.4)	39 (9.0)
Prefer not to identify	91 (0.2)	1 (0.2)
Hispanic, n (%)	2,912 (5.6)	40 (9.8)
^1^Race, n (%)		
White	51,079 (88)	324 (73)
Black	4,746 (8.2)	101 (22.5)
Asian	683 (1.2)	4 (0.9)
Native American/Alaskan	1,421 (2.4)	9 (2.0)
Pacific Islander	120 (0.2)	2 (0.5)
Other	1,289 (2.2)	15 (3.4)
Household Size, M ± SD	2.1 ± 1.1	2.3 ± 1.3
Married, n (%)	36,616 (64)	277 (63)
Income, n (%)		
<$30,000	11,378 (19)	83 (18)
$30,000–59,999	16,754 (29)	116 (26)
$60,000–99,999	13,246 (23)	105 (24)
≥$100,000	9,744 (17)	88 (20)
Missing or prefer not to answer	7,037 (12)	54 (12)
Geographic Area, n (%)		
Northeast	10,127 (17)	116 (26)
Southeast	16,511 (28)	169 (38)
Midwest	11,526 (20)	57 (13)
West	11,842 (21)	61 (13)
Southwest	8,120 (14)	43 (10)
Military Status, n (%)		
Active Duty	55,369 (97.9)	428 (98.2)
Reserves Only	1,153 (2.1)	8 (1.8)
Branch of Military Service, n (%)		
Army	27,936 (48)	240 (54)
Navy	13,400 (23)	71 (16)
Air Force	11,376 (20)	84 (19)
Marine Corps	6,088 (10)	54 (12)
Coast Guard	766 (1.3)	8 (1.8)
National Guard	3,013 (5.2)	15 (3.4)
Merchant Marines	109 (0.2)	0 (0)

^1^ Participants can select more than one race.

The average household size for all survey responders was 2.1 people (SD: 1.1) with 64% reporting being married, compared to a household size of 2.3 (SD: 1.3) and 63% married among the COVID-19 positive responders. Approximately 29% of all responders and 26% of COVID-19 positive responders reported making between $30,000–59,999 per year. Close to 28% of responders reported being from the Southeast (compared to 38% of COVID-19 positives), followed by the West (21%; compared to 13% of COVID-19 positives), the Midwest (20%; compared to 13% of COVID-19 positives), the Northeast (17%; compared to 26% of COVID-19 positives) and the Southwest (14%; compared to 10% of COVID-19 positives). Among all survey responders as well as the self-reported COVID-19 positives, approximately 98% reported their military service as Active Duty while enlisted. The majority of survey responders listed their branch of military service as the Army (48% for all survey responders; 54% for COVID-19 positives), followed by the Navy (23% for all survey responders; 16% for COVID-19 positives) and then the Air Force (20% for all survey responders; 19% for COVID-19 positives).

We compared demographic characteristics between MVP participants who did not respond to the COVID-19 survey with those who did respond. Non-responders and responders differed in age (64.8 vs. 70.8 years, p<0.001), ethnicity (7.5% vs 5.6% Hispanic, p<0.001), and race (88% vs 75%, p<0.001 White and 21% vs. 8% Black, p<0.001), but the two groups did not significantly differ in gender (90% vs. 91%).

Behaviors and public health practices adopted in response to COVID-19 among all survey responders, self-reported COVID-19 positive responders and broken down by gender and age are presented in Table 3. The majority of overall survey responders reported using a face mask or other face covering in public (92% of all responders; 97% of COVID-19 positives), frequent hand washing or use of sanitizer (91% of all responders; 96% of COVID-19 positives) and practicing social distancing (91% of all responders; 95% of COVID-19 positives). After adjustment of gender and age distribution, results from multivariate models indicated that Veterans who reported being COVID-19 positive were significantly more likely to use a face mask or other face covering in public [OR (95%CI): 4.02 (2.07–7.82), p<0.0001], conduct frequent hand washing or use of sanitizer [2.66 (1.58–4.46), p<0.001)] and practice social distancing [2.24 (1.44–3.50), p<0.001]. The majority of Veterans surveyed avoided gatherings of more than 50 people (83% of all responders; 79% of COVID-19 positives), avoided contact with people who could be high-risk for COVID-19 infection (71% for all responders and 71% COVID-19 positives) and avoided eating at restaurants (76% of all responders; 75% of COVID-19 positives) as well as public spaces, gatherings, or crowds (79% for all responders and 78% COVID-19 positives). The frequency of wearing gloves in public was relatively low (35% of all responders; 44% of COVID-19 positives, p < .05), and 56% of all responders and 70% of COVID-19 positives (p < .05) reported cleaning high touch surfaces. Over half of Veterans surveyed (59%) did not cancel their doctor’s appointments during the pandemic. Overall, women were more likely to endorse recommended behaviors and public health practices compared to men. Significant differences were demonstrated among age groups in relation to behaviors and public health practices, but no clear trends were observed.

**Table 3:** Preventive behaviors of MVP COVID-19 Survey responders during COVID-19 pandemic.

Behaviors and Public Health Practices		All Responders	Self-reported Positive		Gender		Age (years)			
			No	Yes	Male	Female	<55	55–64	65–74	≥75
		N = 58,159	N = 13,559	N = 446	N = 52,737	N = 4,865	N = 5,063	N = 8,260	N = 25,292	N = 18,625
Used a face mask or other face covering in public	Prevalence	92.4	91.9	97.4	92.4	93.6	88.6	91.4	92.9	93.6
	OR (95%CI)*		1.0 (Ref.)	4.02* (2.07–7.82)	1.0 (Ref.)	1.62* (1.22–2.14)	0.74* (0.57–0.95)	0.94 (0.76–1.15)	1.04 (0.91–1.20)	1.0 (Ref.)
Used gloves in public	Prevalence	35.3	34.8	43.9	35.1	37.8	32.7	36.9	36.6	33.6
	OR (95%CI)*		1.0 (Ref.)	1.44* (1.18–1.75)	1.0 (Ref.)	1.13 (0.99–1.29)	1.04 (0.89–1.21)	1.23* (1.10–1.38)	1.21* (1.12–1.38)	1.0 (Ref.)
Washed your hands with soap or used hand sanitizer several times a day	Prevalence	91.4	90.5	96.2	91.3	94.0	92.8	91.7	91.6	91.2
	OR (95%CI)*		1.0 (Ref.)	2.66* (1.58–4.46)	1.0 (Ref.)	1.45* (1.11–1.89)	1.22 (0.93–1.59)	1.31* (1.07–1.61)	1.06 (0.93–1.21)	1.0 (Ref.)
Cleaned high touch surfaces like door handles, counters, faucets, and remote controls	Prevalence	56.1	56.1	69.5	54.9	70.0	66.2	63.2	56.7	50.1
	OR (95%CI)*		1.0 (Ref.)	1.59* (1.29–1.97)	1.0 (Ref.)	1.80* (1.56–2.09)	2.07* (1.78–2.42)	1.83* (1.63–2.05)	1.45* (1.34–1.56)	1.0 (Ref.)
Practiced social distancing (avoiding contact with anyone outside of the home)	Prevalence	90.7	89.0	94.8	90.7	92.1	88.1	91.2	91.3	91.0
	OR (95%CI)*		1.0 (Ref.)	2.24* (1.44–3.50)	1.0 (Ref.)	1.37* (1.08–1.73)	0.91 (0.72–1.14)	1.24* (1.03–1.50)	1.16* (1.03–1.32)	1.0 (Ref.)
Avoided contact with people who could be high-risk	Prevalence	70.8	70.1	71.4	70.5	75.7	72.2	72.1	71.2	69.8
	OR (95%CI)*		1.0 (Ref.)	1.01 (0.82–1.26)	1.0 (Ref.)	1.45* (1.24–1.70)	1.30* (1.10–1.53)	1.26* (1.12–1.43)	1.13* (1.04–1.23)	1.0 (Ref.)
Avoided eating at restaurants	Prevalence	76.0	73.7	75.1	75.7	80.4	72.7	75.2	76.7	76.7
	OR (95%CI)*		1.0 (Ref.)	1.07 (0.85–1.34)	1.0 (Ref.)	1.65* (1.40–1.94)	0.92 (0.78–1.08)	0.96 (0.85–1.09)	1.06 (0.97–1.16)	1.0 (Ref.)
Avoided public spaces, gatherings, or crowds	Prevalence	78.6	77.5	78.4	78.3	83.2	74.7	77.0	78.8	80.5
	OR (95%CI)*		1.0 (Ref.)	1.09 (0.85–1.38)	1.0 (Ref.)	1.52* (1.28–1.80)	0.78* (0.66–0.92)	0.95 (0.83–1.09)	0.96 (0.88–1.06)	1.0 (Ref.)
Avoided gatherings of more than 50 people	Prevalence	82.9	80.2	79.3	82.6	87.4	81.6	82.3	83.4	83.3
	OR (95%CI)*		1.0 (Ref.)	0.93 (0.73–1.19)	1.0 (Ref.)	1.64* (1.36–1.99)	1.04 (0.86–1.25)	1.14 (0.99–1.31)	1.09 (0.99–1.20)	1.0 (Ref.)
Cancelled doctor’s appointments	Prevalence	41.1	38.9	44.1	40.8	45.7	40.6	40.2	41.9	40.7
	OR (95%CI)*		1.0 (Ref.)	1.25* (1.03–1.51)	1.0 (Ref.)	1.43* (1.25–1.63)	1.04 (0.90–1.20)	1.03 (0.92–1.15)	1.10* (1.01–1.18)	1.0 (Ref.)

Abbreviations: OD (95%CI): Odds Ratio (95% Confident Interval); Ref.: reference group.

*P<0.05, mutually adjustment of gender (male, female, missing), age (years: <55, 55–64, 65–74, 75+, missing) and self-reported COVID positive (no, yes, non-response) applying logistic regression model; number of missing of gender: 557; missing of age: 919; and non-response of self-reported status of COVID-19: 44,154.

Changes in various aspects of life for all survey respondents are reported in Table 4. Regarding aspects of life that changed as a result of the pandemic, over half of respondents reported no change in the amount of physical activity (55% compared to 32% who reported a decrease), time spent talking to family/friends (57% compared to 23% who reported a decrease), amount slept (79% compared to 10% who reported a decrease), time watching TV/streaming service (51% compared to 41% who reported an increase), time spent on hobbies (55%), amount eaten (69%), and practicing relaxation techniques (52%). The amount of money spent was relatively similar between those reporting staying the same (42%) and 44% reporting a decrease, with 12% of respondents reporting an increase. Responses for amount of alcohol drank indicated 42% reporting no change, 8% reporting a decrease, 5% an increase and over 25,000 respondents (45%) reported not applicable. Responses for the amount smoking/vaping demonstrated 11% reporting no change, with 85% reporting not applicable. Thirty-two percent of respondents reported no change in the amount worked from home (with 51% reporting not applicable) and 46% reported no change in time spent on social media (31% reported not applicable). The majority of respondents (71%) reported not applicable for time spent playing video games.

**Table 4:** Lifestyle behaviors of MVP COVID-19 Survey responders during COVID-19 pandemic.

Since the COVID-19 pandemic started, have any of the following aspects in your life change? n (%):				
	Decreased	Stayed the same	Increased	Not applicable
Amount of money you’ve spent	25,298 (44.3)	23,854 (41.8)	6,915 (12.1)	1,007 (1.8)
Amount of physical activity	17,937 (31.8)	31,270 (55.4)	5,885 (10.4)	1,351 (2.4)
Time spent talking to family/friends	12,651 (22.5)	32,109 (57.1)	9,665 (17.2)	1,778 (3.2)
Time spent talking to work colleagues	7,826 (14)	10,599 (18.9)	1,298 (2.3)	36,331 (64.8)
Number of hours you work in usual workplace	6,679 (11.9)	10,967 (19.5)	1,613 (2.9)	37,050 (65.8)
Amount you sleep	5,613 (9.8)	45,046 (78.8)	5,093 (8.9)	1,409 (2.5)
Amount of alcohol you drink	4,198 (7.5)	23,727 (42.3)	2,822 (5)	25,281 (45.1)
Amount you smoke/vape	1,088 (1.9)	6,240 (11.2)	1,037 (1.9)	47,591 (85.1)
Time spent reading or listening to the news	3,581 (6.3)	28,560 (49.9)	23,742 (41.5)	1,316 (2.3)
Time watching TV/streaming service	2,260 (4)	29,221 (51.4)	23,537 (41.4)	1,791 (3.2)
Time spent doing hobbies/things you enjoy	8,424 (15)	30,849 (54.8)	11,026 (19.6)	5,949 (10.6)
Amount you eat	6,522 (11.5)	39,183 (69.3)	10,133 (17.9)	727 (1.3)
Time spent on social media	3,439 (6.2)	25,380 (45.5)	9,965 (17.9)	17,006 (30.5)
Number of hours you work at home	2,358 (4.2)	18,304 (32.3)	7,339 (13)	28,639 (50.6)
Practicing relaxation/mindfulness/meditation	2,562 (4.6)	28,835 (51.7)	6,895 (12.4)	17,475 (31.3)
Time spent playing video games	1,238 (2.2)	10,621 (19)	4,135 (7.4)	39,992 (71.4)

## Discussion

This paper describes the development, deployment, and initial findings from the MVP COVID-19 Survey. Development of the MVP COVID-19 Survey provides a unique opportunity to assess multiple aspects of COVID-19 over time with the flexibility of multiple modalities, including online and paper options. For the first phase of data collection, the overall survey participation rate was 8%, although this varied widely across survey dissemination method. Completion rates were considerably higher among Veterans who were mailed the paper version of the survey (ranging from 36–39%) compared with those asked to complete the survey online (ranging from 6–8%). Nevertheless, development of online survey completion for MVP offers an enhanced method of collecting self-report data from the cohort, particularly among younger Veterans. Based on these response rates, the second phase of data collection adopted the mail-based approach for re-contact of MVP COVID-19 Survey non-responders starting in the Fall of 2021. Future work will provide details regarding this data collection effort, and provide overall characteristics for all respondents, comparing multiple modalities of data capture. Additionally, public health behaviors related to COVID-19 at various time points throughout the pandemic will be examined.

The MVP COVID-19 Survey provides a systematic collection of data regarding COVID-19 behaviors among Veterans and represents the first large-scale, national surveillance efforts of COVID-19 in the Veteran population. Those who completed the survey were slightly older than the overall MVP population (70.8 vs 64.4 years old [14]) but the demographic characteristics of the MVP COVID-19 Survey responders were more diverse, allowing for greater representation of Veteran data on COVID-19 exposure, health, and behavioral practices. In general, Veterans who utilize VHA services tend to be older, white, and male [24]. The demographic characteristics of initial MVP COVID-19 responders suggest generalizability to the broader VHA population.

Among respondents who reported testing positive for COVID-19, we observed a higher response among Black and Hispanic Veterans compared to Non-Hispanic White Veterans in the first wave of survey dissemination. This is consistent with studies and reports suggesting that Black and Hispanic communities have increased rates of infection and hospitalization compared to White counterparts [25–27]. Combined with the increased diversity among the MVP cohort, collecting information on exposure status, diagnosis, medical interventions, concurrent comorbidities and the impact of the virus on behavior and well-being allows for future work examining self-reported COVID-19 data combined with health record data to better understand the racial and ethnic differences in COVID-19 among Veterans.

The prevalence of Veterans wearing masks and practicing social distancing demonstrates compliance of public health recommendations surrounding the pandemic, especially with research reporting facial masks and social distancing helping to reduce and prevent the spread of COVID-19 [28, 29]. Among MVP participants, 92% reported using a face mask or other face covering in public, higher than the 62–77% reported from a representative sample of the general US population between April and May of 2020 [8]. A major reason for this difference could be older age of the MVP cohort, which has been previously associated with higher reported rates of wearing cloth face coverings in public [9]. Generally, the COVID-19 positive respondents demonstrated higher reporting of engaging in protective behaviors. This could be a result of increased behavior following infection, or indicative of increased exposure through vocational or other activity. Women were more likely to endorse recommended public health practices, consistent with research demonstrating women are more likely to adopt measures designed to reduce the risk of COVID-19 [30]. Clear differences were not reported across age groups regarding public health behaviors. Behavioral data collected through the survey can inform guidelines for community prevention to reduce the spread of COVID-19 infection. Future work will explore changes and trends over time in responses related to COVID-19 behavioral practices.

Changes in aspects of life resulting from COVID-19, for the most part, were not reported by respondents. Increases in alcohol usage and tobacco as a result of the pandemic have been reported [31], however, MVP survey respondents primarily reported no increase or not applicable. This is consistent with two survey studies of Veterans which found that alcohol and tobacco use rates stayed the same or declined, at least in the early stages of the pandemic [11, 12]. Additionally, items related to smoking and work practices were largely responded to as not applicable, likely given the higher age of respondents. For example, work-related activities (interacting with colleagues, hours spent in workplace, and hours worked at home) were selected as not applicable, possibly indicative of a large proportion of retired individuals among MVP. Future work will explore the tangible and intangible effects of loss of resources as a result of the pandemic, along with mental health outcomes associated with the pandemic.

Limitations of the current research include the voluntary nature of participation in MVP overall, as well as among MVP COVID-19 Survey respondents. Participants may be reflective of a self-selection bias, which limits the generalizability to the VHA Veteran community and beyond. Future work examining the overall MVP COVID-19 Survey data collection effort will provide comparison to the MVP cohort and VHA Veterans for enhanced inference. An additional limitation is that the self-reported COVID-19 diagnosis question (question 29) was missing for many participants and the number of COVID-19 positive participants was quite small. This question could also be enhanced with the addition of “Yes, suspected by self and/or others but not confirmed by a test” as a response option. Continued assessment of COVID-19 among the MVP cohort will work to update this question. Future efforts will include validating self-reported COVID-19 diagnosis with lab confirmed test results from the VA EHR.

Strengths of the MVP COVID-19 Survey include development of a comprehensive tool for disease surveillance, identifying possible comorbid disease-specific subgroups at risk, and tracking trends to ascertain the impact of COVID-19 on Veterans. Our systematic and structured data collection method embedded within the MVP framework provides opportunity for rapid-response and seamless data integration capability with pre-existing MVP information collected from MVP Baseline and MVP Lifestyle Surveys [14] in addition to the VA EHR. This linkage of extensive longitudinal data on COVID-19 is one of the strengths of the MVP mega-biobank. Such wide-ranging data coverage and the large scale of the VA population provide unprecedented opportunities for new discoveries in both biomedical research and infrastructure development. The novel information captured from the MVP COVID-19 Survey also provides a useful resource to validate true case rates for COVID-19 testing and mortality. The MVP COVID-19 data combined with Veterans’ health record information, responses to MVP surveys, as well as genetic data can enable MVP researchers to identify new targets for disease prevention, treatment, and management with an emphasis on variability in an individual’s genes, environment, and lifestyle.

## Conclusion

The MVP COVID-19 Survey provides a systematic collection of data regarding COVID-19 behaviors among Veterans and represents the first large-scale, national surveillance efforts of COVID-19 in the Veteran population. Continued work will examine the overall response to the survey with comparison to available VA health record data.

## Supporting information

S1 Appendix MVP COVID-19 Survey. (PDF)

S1 File Million Veteran Program full acknowledgments. (DOCX)
