# Supplementary material for: Million Veteran Program’s response to COVID-19: Survey development and preliminary findings
Source: PLoS One. 2022 Apr 25;17(4):e0266381. doi: 10.1371/journal.pone.0266381 (PMC9037905; doi:10.1371/journal.pone.0266381)
Supplement: S1 Appendix — (PDF) [file pone.0266381.s001.pdf]

Section A: Demographics

1. What is today's date?

☐☐ / ☐☐ / ☐☐☐☐
mm dd yyyy

2. What is your date of birth?

☐☐ / ☐☐ / ☐☐☐☐
mm dd yyyy

3. What is your gender?

- ☐ Male
- ☐ Prefer not to answer
- ☐ Female

4. Are you Spanish, Hispanic, or Latino?

- ☐ No, not Spanish, Hispanic, Latino
- ☐ Yes, Mexican, Mexican American, Chicano
- ☐ Yes, Puerto Rican
- ☐ Yes, Cuban
- ☐ Yes, other Spanish, Hispanic, Latino

5. What is your race? *(Mark all that apply)*

- ☐ White
- ☐ Black / African - American
- ☐ American Indian / Alaska Native
- ☐ Chinese
- ☐ Japanese
- ☐ Asian Indian
- ☐ Other Asian
- ☐ Filipino
- ☐ Pacific Islander
- ☐ Other

6. What is your highest degree or level of school you have completed?

- ☐ Less than high school
- ☐ High school diploma / GED
- ☐ Some college credit, but no degree
- ☐ Associate's degree (e.g., AA, AS)
- ☐ Bachelor's degree (e.g., BA, BS)
- ☐ Master's degree (e.g., MA, MS, MBA)
- ☐ Professional or Doctorate degree

7. What is your current marital status?

- ☐ Married
- ☐ Divorced
- ☐ Civil commitment
- ☐ Widowed
- ☐ Cohabitating
- ☐ Never married
- ☐ Separated

8. Including yourself, how many people currently live in your household?

1	2	3	4	5	6	7	8	9+
☐	☐	☐	☐	☐	☐	☐	☐	☐

9. Which income category represents the total income of your household from all sources (before taxes and deductions) during the last 12 months?

- ☐ Less than $10,000
- ☐ $10,000 - $19,999
- ☐ $20,000 - $29,999
- ☐ $30,000 - $39,999
- ☐ $40,000 - $49,999
- ☐ $50,000 - $59,999
- ☐ $60,000 - $74,999
- ☐ $75,000 - $99,999
- ☐ $100,000 - $149,999
- ☐ $150,000 or more
- ☐ Prefer not to answer

10. What is your height:

☐ feet ☐☐ inches

11. What is your weight:

☐☐☐ pounds

12. In which branch of the service did you serve? *(Mark all that apply)*

- ☐ Army
- ☐ Navy
- ☐ Air Force
- ☐ Coast Guard
- ☐ Marine Corps
- ☐ National Guard
- ☐ Merchant Marines
- ☐ NOAA
- ☐ Public Health Service
- ☐ None *(Skip to Qu. 15)*

13. Please indicate whether your service was:

- ☐ Active Duty
- ☐ Reserves Only
- ☐ Not Applicable (Not in the military)

14. When did you serve? *(Mark all that apply)*

- ☐ September 2001 or later
- ☐ August 1990 to August 2001 (includes Gulf War)
- ☐ May 1975 to July 1990
- ☐ August 1964 to April 1975 (Vietnam era)
- ☐ February 1955 to July 1964
- ☐ July 1950 to January 1955 (Korean War)
- ☐ January 1947 to June 1950
- ☐ December 1941 to December 1946 (WWII)
- ☐ November 1941 or earlier

15. How often do you have a drink containing alcohol?

- ☐ Never *(Skip to Qu. 18)*
- ☐ 1 – 3 days per month
- ☐ 1 day per week
- ☐ 2 – 3 days per week
- ☐ 4 – 5 days per week
- ☐ 6 or more days per week

16. How many drinks containing alcohol do you have on a typical day when you are drinking?

- ☐ 1 or 2
- ☐ 3 or 4
- ☐ 5 or 6
- ☐ 7 to 9
- ☐ 10 or more

17. How often do you have six or more drinks on one occasion?

- ☐ Never
- ☐ Less than monthly
- ☐ Monthly
- ☐ 2 – 3 times per week
- ☐ 4 or more times per week

18. In your lifetime have you smoked a total of at least 100 cigarettes, cigars, or pipes?

- ☐ Yes
- ☐ No → *(Skip to Qu. 21)*

19. Have you ever smoked daily or almost every day for at least one year?

- ☐ Yes
- ☐ No

20. Do you smoke now?

- ☐ Yes, daily
- ☐ Yes, occasionally
- ☐ Not at all

The following questions concern electronic vaping products for nicotine use. Do not include marijuana use.

21. Have you ever used an e-cigarette or other electronic vaping product, even just one time, in your entire life?

- ☐ Yes
- ☐ No *(Skip to Qu. 23)*
- ☐ Prefer not to answer *(Skip to Qu. 23)*
- ☐ Don’t know *(Skip to Qu. 23)*

22. Do you NOW use e-cigarettes or other electronic vaping products every day, some days, or not at all?

- ☐ Every day
- ☐ Some days
- ☐ Not at all
- ☐ Prefer not to answer
- ☐ Don’t know

Section B: COVID-19 Exposure/Household Contact

23. Have you been in close contact with anyone with COVID-19 like symptoms?

- ☐ Yes, I was in contact with a person with COVID-19 who was confirmed positive by a test
- ☐ Yes, I was in contact with a person with COVID-19 symptoms, but was not confirmed by a test
- ☐ No, not to my knowledge

24. Has anyone in your household had COVID-19? *Please do not include yourself.*

- ☐ Yes → Please indicate the number of people. ☐ People
- ☐ No

25. Are you a healthcare worker helping to manage patients with COVID-19?

- ☐ Yes
- ☐ No
- ☐ Don't know
- ☐ Prefer not to answer

Section C: COVID-19 Symptoms/Diagnosis

26. Have you experienced any of the following symptoms (more than normal) since January 2020? Please check "Yes" or "No" next to each symptom.

If yes, please indicate the number of days you experienced any of these symptoms.	No	Yes	Number of Days You Experienced Symptom
a. Coughing a lot for more than an hour, or 3 or more coughing episodes in 24 hours	☐	☐ →	☐☐☐
b. Shortness of breath	☐	☐ →	☐☐☐
c. Unusual chest pain or tightness in your chest	☐	☐ →	☐☐☐
d. Fatigue (struggling to get out of bed)	☐	☐ →	☐☐☐
e. Feeling of heaviness in arms or legs	☐	☐ →	☐☐☐
f. Headache	☐	☐ →	☐☐☐
g. Loss of sense of smell or taste	☐	☐ →	☐☐☐
h. Sore throat	☐	☐ →	☐☐☐
i. Diarrhea, nausea and/or vomiting	☐	☐ →	☐☐☐
j. Fever/chills (temp>100.4 Fahrenheit)	☐	☐ →	☐☐☐

27. Did you seek advice from a healthcare professional for these symptoms?

- ☐ Yes → a. If yes, where did you seek care? *(Mark all that apply)*
  - ☐ VA facility
  - ☐ Doctor's office
  - ☐ Emergency department
  - ☐ Telemedicine/telephone triage
  - ☐ Health department/public health clinic
  - ☐ Retail clinic/pharmacy
  - ☐ Urgent care
  - ☐ Other

  b. If yes, how long after your symptoms started did you seek care?
  - ☐ Less than 2 days
  - ☐ 2 - 7 days
  - ☐ Greater than 1 week
- ☐ No *(Skip to Qu. 41)*

28. Did doctors use a laboratory test to check that you didn't have influenza (Flu)?

- ☐ Yes
- ☐ No
- ☐ Don't know

29. Have you been diagnosed with COVID-19?

- ☐ Yes, confirmed by a positive laboratory test
- ☐ Yes, suspected by a doctor but not confirmed by a test *(Skip to Qu. 41)*
- ☐ No *(Skip to Qu. 41)*

30. Do you know what type of laboratory test you had for COVID-19?

- ☐ Yes, by nasal swab (PCR)
- ☐ Yes, by blood test (antibody)
- ☐ Yes, by another test
- ☐ Don't know

31. Is there a suspected source of your COVID-19?

- ☐ Travel related
- ☐ Family member was sick
- ☐ Coworker or other work contact was sick
- ☐ Friend or other social contact was sick
- ☐ Don't know
- ☐ Prefer not to answer

Section D: COVID-19 Hospitalization/Treatment

32. Were you hospitalized for treatment of COVID-19?

☐ Yes
☐ No *(Skip to Qu. 38)*

33. Were you hospitalized at a VA facility?

☐ Yes
☐ No

34. What date were you admitted to the hospital for treatment of COVID-19?

☐☐ / ☐☐ / ☐☐☐☐
mm dd yyyy

35. What date were you discharged from the hospital after treatment of COVID-19?

☐☐ / ☐☐ / ☐☐☐☐
mm dd yyyy

36. Did you require a breathing tube through the mouth for respiratory support while in the hospital (intubation / mechanical ventilation / respirator)?

☐ Yes
☐ No

37. Were you hospitalized in an Intensive Care Unit (ICU) for treatment of COVID-19?

☐ Yes
☐ No
☐ Don't know

38. Do you know if doctors used any of the following medications to treat your illness while you were sick with COVID-19? *(Mark all that apply)*

☐ Tamiflu (oseltamivir) or Xofluza (baloxavir marboxil)
☐ Chloroquine or Hydroxychloroquine
☐ Azithromycin
☐ Kevzara (sarilumab), Actemra (tocilizumab), Sylvant (siltuximab) or another IL6 pathway blocker medication
☐ Baricitinib (Olumiant), Ruxolitinib (Jakafi), tofacitinib (XELJANZ), fedratinib (Inrebic) or another JAK inhibitor medication
☐ Remdesivir (GS-5735)
☐ Darunavir (Prezista), Lopinavir/Ritonavir (Kaletra) or another protease inhibitor
☐ Favipiravir (Avigan)
☐ Sofosbuvir or ribavirin
☐ None of the above
☐ Don't know

39. Did you receive respiratory support at home to treat your COVID-19, such as oxygen therapy by nasal prong or facemask or CPAP machine?

☐ Yes
☐ No ⇒ *(Skip to Qu. 41)*

40. If yes, for how long did you need respiratory support at home? *Please enter the duration of your respiratory support in days.*

☐ days

Section E: COVID-19 Impact Behavior/Well-Being

The next questions ask about your behaviors and well-being since the COVID-19 pandemic and the impact it has had on you.

41. Which of the following have you done since the COVID-19 pandemic? *(Mark all that apply)*

- ☐ Used a face mask or other face covering while in public
- ☐ Used gloves while in public
- ☐ Washed your hands with soap or used hand sanitizer several times a day
- ☐ Cleaned high touch surfaces like door handles, counters, faucets, and remote controls
- ☐ Practiced social distancing (avoiding contact with anyone outside of the home)
- ☐ Avoided contact with people who could be high-risk
- ☐ Avoided eating at restaurants
- ☐ Avoided public spaces, gatherings, or crowds
- ☐ Avoided gatherings of more than 50 people
- ☐ Cancelled doctor's appointments

42. Since the COVID-19 pandemic started, have any of the following aspects of your life changed?

		Decreased	Stayed the Same	Increased	Not Applicable
a.	**Amount you sleep**	☐	☐	☐	☐
b.	**Amount of physical activity you do**	☐	☐	☐	☐
c.	**Amount you smoke/vape**	☐	☐	☐	☐
d.	**Amount of alcohol you drink**	☐	☐	☐	☐
e.	**Number of hours you work in usual workplace**	☐	☐	☐	☐
f.	**Number of hours you work at home**	☐	☐	☐	☐
g.	**Time spent talking to family/friends**	☐	☐	☐	☐
h.	**Time spent talking to work colleagues**	☐	☐	☐	☐
i.	**Practicing relaxation / mindfulness / meditation**	☐	☐	☐	☐
j.	**Time watching TV/streaming services**	☐	☐	☐	☐
k.	**Time spent reading or listening to the news**	☐	☐	☐	☐
l.	**Time spent on social media**	☐	☐	☐	☐
m.	**Time spent playing video games**	☐	☐	☐	☐
n.	**Time spent doing hobbies/things you enjoy**	☐	☐	☐	☐
o.	**Amount you eat**	☐	☐	☐	☐
p.	**Amount of money you've spent**	☐	☐	☐	☐

43. Over the past 2 weeks, have you been bothered by any of these problems?

		Not at all	Several days	More days than not	Nearly every day
a.	**Feeling nervous, anxious, or on edge**	☐	☐	☐	☐
b.	**Not being able to stop or control worrying**	☐	☐	☐	☐
c.	**Feeling down, depressed, or hopeless**	☐	☐	☐	☐
d.	**Little interest or pleasure in doing things**	☐	☐	☐	☐

44. Since the COVID-19 pandemic, for each of the statements below please select the best choice that describes how you feel. *Select only one response for each question or statement*.

		Never	Rarely	Sometimes	Usually	Always	Don't know or N/A
**Social Isolation**							
a.	**I feel left out…**	☐	☐	☐	☐	☐	☐
b.	**I feel that people barely know me…**	☐	☐	☐	☐	☐	☐
c.	**I feel isolated from others…**	☐	☐	☐	☐	☐	☐
d.	**I feel that people are around me, but not with me…**	☐	☐	☐	☐	☐	☐

45. Since the COVID-19 pandemic, for each of the statements below please select the best choice that describes how you feel. *Select only one response for each question or statement*.

		Never	Rarely	Sometimes	Usually	Always	Don't know or N/A
**Emotional Support**							
a.	**I have someone who will listen to me when I need to talk.**	☐	☐	☐	☐	☐	☐
b.	**I have someone to confide in or talk to about myself or my problems.**	☐	☐	☐	☐	☐	☐
c.	**I have someone who makes me feel appreciated.**	☐	☐	☐	☐	☐	☐
d.	**I have someone to talk with when I have a bad day.**	☐	☐	☐	☐	☐	☐

46. Since the COVID-19 pandemic, for each of the items below please select the best choice describing the degree of impact. *Select only one response for each question or statement.*

		No Loss	Minimal Loss	Noticeable Loss	Extreme Loss	Don't Know or N/A
a.	**Adequate food**	☐	☐	☐	☐	☐
b.	**Your residence / home you live in**	☐	☐	☐	☐	☐
c.	**Things you need for your children or members of your household**	☐	☐	☐	☐	☐
d.	**Money for extras**	☐	☐	☐	☐	☐
e.	**Savings or emergency money**	☐	☐	☐	☐	☐
f.	**Adequate income**	☐	☐	☐	☐	☐
g.	**Financial credit**	☐	☐	☐	☐	☐
h.	**Your retirement security**	☐	☐	☐	☐	☐
i.	**Free time**	☐	☐	☐	☐	☐
j.	**Time for enough sleep**	☐	☐	☐	☐	☐
k.	**Feeling valuable to other people**	☐	☐	☐	☐	☐
l.	**A feeling of intimacy with one or more family members**	☐	☐	☐	☐	☐
m.	**The feeling that you're accomplishing the goals in your life**	☐	☐	☐	☐	☐
n.	**Time with your loved ones**	☐	☐	☐	☐	☐
o.	**The sense of a daily routine**	☐	☐	☐	☐	☐
p.	**Health of a family member / friend**	☐	☐	☐	☐	☐
q.	**Stable employment**	☐	☐	☐	☐	☐
r.	**Ability to organize tasks**	☐	☐	☐	☐	☐
s.	**Time needed to do your work**	☐	☐	☐	☐	☐
t.	**Understanding from your boss**	☐	☐	☐	☐	☐
u.	**Support from your co-workers**	☐	☐	☐	☐	☐
v.	**The chance to get more training or education**	☐	☐	☐	☐	☐

	Continued	No Loss	Minimal Loss	Noticeable Loss	Extreme Loss	Don't Know or N/A
w.	**Feeling of being independent**	☐	☐	☐	☐	☐
x.	**Companionship with others**	☐	☐	☐	☐	☐
y.	**Feeling that your life has meaning or purpose**	☐	☐	☐	☐	☐
z.	**Involvement with your church**	☐	☐	☐	☐	☐
aa.	**Help with tasks at home**	☐	☐	☐	☐	☐
bb.	**Loyalty of friends**	☐	☐	☐	☐	☐
cc.	**Help with childcare**	☐	☐	☐	☐	☐
dd.	**Involvement in organizations or clubs**	☐	☐	☐	☐	☐

Section F: Medical Conditions/Comorbidity

47. **We'd like to ask about your general health. Please tell us if you have ever been diagnosed with the following conditions. Check the appropriate box and indicate the year of diagnosis and whether you currently take any medication(s) ("TAKE MEDS") for that condition.** *(Mark all that apply)*

Circulatory System Problems	YES	YEAR DIAGNOSED	TAKE MEDS
High blood pressure (Hypertension)	☐	☐☐☐☐	☐
Stroke	☐	☐☐☐☐	☐
Transient ischemic attack (TIA)	☐	☐☐☐☐	☐
Heart attack	☐	☐☐☐☐	☐
Coronary artery / Coronary heart disease (includes angina)	☐	☐☐☐☐	☐
Peripheral vascular disease	☐	☐☐☐☐	☐
High cholesterol	☐	☐☐☐☐	☐
Pulmonary embolism or deep vein thrombosis (DVT)	☐	☐☐☐☐	☐
Congestive heart failure	☐	☐☐☐☐	☐
Other circulatory system problem	☐	☐☐☐☐	☐

Mental Health Disorders	YES	YEAR DIAGNOSED	TAKE MEDS
Anxiety reaction / Panic disorder	☐	☐☐☐☐	☐
Attention deficit hyper-activity disorder (ADHD)	☐	☐☐☐☐	☐
Bipolar disorder	☐	☐☐☐☐	☐
Post traumatic stress disorder (PTSD)	☐	☐☐☐☐	☐
Depression	☐	☐☐☐☐	☐
Eating disorder	☐	☐☐☐☐	☐
Personality disorder	☐	☐☐☐☐	☐
Schizophrenia	☐	☐☐☐☐	☐
Social phobia	☐	☐☐☐☐	☐
Other mental health disorder	☐	☐☐☐☐	☐

Skeletal / Muscular Problems	YES	YEAR DIAGNOSED	TAKE MEDS
Osteoarthritis	☐	☐☐☐☐	☐
Rheumatoid arthritis	☐	☐☐☐☐	☐
Other arthritis	☐	☐☐☐☐	☐
Gout	☐	☐☐☐☐	☐
Osteoporosis	☐	☐☐☐☐	☐
Other skeletal / muscular problem	☐	☐☐☐☐	☐

Hearing / Vision	YES	YEAR DIAGNOSED	TAKE MEDS
Cataracts	☐	☐☐☐☐	☐
Glaucoma	☐	☐☐☐☐	☐
Macular degeneration	☐	☐☐☐☐	☐
Blindness, all causes	☐	☐☐☐☐	☐
Tinnitus or ringing in the ears	☐	☐☐☐☐	☐
Severe hearing loss or partial deafness in one or both ears	☐	☐☐☐☐	☐

Infectious Diseases

	YES	YEAR DIAGNOSED	TAKE MEDS
Tuberculosis	☐	☐☐☐☐	☐
Hepatitis C	☐	☐☐☐☐	☐
HIV / AIDS	☐	☐☐☐☐	☐
Other infectious disease	☐	☐☐☐☐	☐

Kidney Disease

	YES	YEAR DIAGNOSED	TAKE MEDS
Kidney disease without dialysis	☐	☐☐☐☐	☐
Kidney disease with dialysis	☐	☐☐☐☐	☐
Acute kidney disease with no current dialysis	☐	☐☐☐☐	☐

Digestive System Problems

	YES	YEAR DIAGNOSED	TAKE MEDS
Acid reflux / GERD	☐	☐☐☐☐	☐
Peptic ulcers	☐	☐☐☐☐	☐
Bowel obstruction	☐	☐☐☐☐	☐
Colon polyps	☐	☐☐☐☐	☐
Irritable bowel syndrome (IBS)	☐	☐☐☐☐	☐
Ulcerative colitis	☐	☐☐☐☐	☐
Crohn's disease	☐	☐☐☐☐	☐
Celiac disease / Sprue	☐	☐☐☐☐	☐
Other digestive system disorder	☐	☐☐☐☐	☐

Cancer

	YES	YEAR DIAGNOSED	TAKE MEDS
Breast cancer	☐	☐☐☐☐	☐
Colon cancer / Rectal cancer	☐	☐☐☐☐	☐
Lung cancer	☐	☐☐☐☐	☐
Prostate cancer	☐	☐☐☐☐	☐
Skin cancer	☐	☐☐☐☐	☐
Other cancer	☐	☐☐☐☐	☐

Nervous System Problems

	YES	YEAR DIAGNOSED	TAKE MEDS
Migraine headaches	☐	☐☐☐☐	☐
Other headaches	☐	☐☐☐☐	☐
Memory loss or impairment	☐	☐☐☐☐	☐
Dementia (includes Alzheimer's, vascular, etc.)	☐	☐☐☐☐	☐
Concussion or loss of consciousness	☐	☐☐☐☐	☐
Traumatic brain injury	☐	☐☐☐☐	☐
Spinal cord injury or impairment	☐	☐☐☐☐	☐
Epilepsy / Seizure	☐	☐☐☐☐	☐
Parkinson's disease	☐	☐☐☐☐	☐
Amyotrophic lateral sclerosis (Lou Gehrig's disease)	☐	☐☐☐☐	☐
Multiple sclerosis	☐	☐☐☐☐	☐
Other nervous system problem	☐	☐☐☐☐	☐

Other Conditions

	YES	YEAR DIAGNOSED	TAKE MEDS
Asthma	☐	☐☐☐☐	☐
Chronic lung disease (COPD, Emphysema or Bronchitis)	☐	☐☐☐☐	☐
Diabetes / “sugar”	☐	☐☐☐☐	☐
Enlarged prostate (Benign prostatic hyperplasia)	☐	☐☐☐☐	☐
Liver condition (e.g., Cirrhosis)	☐	☐☐☐☐	☐
Skin condition (e.g., Eczema, Psoriasis)	☐	☐☐☐☐	☐
Sleep apnea	☐	☐☐☐☐	☐
Thyroid problems	☐	☐☐☐☐	☐
Gulf War Illness/ Syndrome	☐	☐☐☐☐	☐
Chronic Fatigue Syndrome	☐	☐☐☐☐	☐
Fibromyalgia	☐	☐☐☐☐	☐
Other disease / disorder	☐	☐☐☐☐	☐

48. Did you receive the following vaccines while in the military? *If yes, please write in the year of the last vaccine dose.*

Anthrax

- ☐ Yes → Year Vaccinated: ☐☐☐☐
- ☐ No
- ☐ Don't Know

Small Pox

- ☐ Yes → Year Vaccinated: ☐☐☐☐
- ☐ No
- ☐ Don't Know

Rabies

- ☐ Yes → Year Vaccinated: ☐☐☐☐
- ☐ No
- ☐ Don't Know

Yellow Fever

- ☐ Yes → Year Vaccinated: ☐☐☐☐
- ☐ No
- ☐ Don't Know

Typhoid

- ☐ Yes → Year Vaccinated: ☐☐☐☐
- ☐ No
- ☐ Don't Know

Japanese Encephalitis

- ☐ Yes → Year Vaccinated: ☐☐☐☐
- ☐ No
- ☐ Don't Know

49. In general, would you say your health is:

- ☐ Excellent
- ☐ Very Good
- ☐ Good
- ☐ Fair
- ☐ Poor

50. In the PAST YEAR, have you received health care that was paid for by any of the following insurance types? *(Mark all that apply)*

- ☐ Private insurance
- ☐ TRICARE
- ☐ Medicare
- ☐ Medicaid
- ☐ Veterans Choice Program
- ☐ VA health care
- ☐ Indian Health

51. In the PAST YEAR, about how much of your health care did you get at a VA facility (e.g., doctor's visits, hospitalizations, urgent care visits, or counseling)?

- ☐ None
- ☐ 1 – 25%
- ☐ 26 – 50%
- ☐ 51 – 75%
- ☐ 76 – 99%
- ☐ 100%

52. In the PAST YEAR, how many times were you a patient in a hospital overnight or longer?

VA Facility

- ☐ None
- ☐ 1 - 3
- ☐ 4 - 6
- ☐ 7 - 9
- ☐ 10 or more

Non-VA Healthcare Facility

- ☐ None
- ☐ 1 - 3
- ☐ 4 - 6
- ☐ 7 - 9
- ☐ 10 or more

53. How many <u>prescription</u> medications do you currently receive from:

VA Pharmacy

- ☐ None
- ☐ 1 - 3
- ☐ 4 - 6
- ☐ 7 - 9
- ☐ 10 or more

Non-VA Pharmacy

- ☐ None
- ☐ 1 - 3
- ☐ 4 - 6
- ☐ 7 - 9
- ☐ 10 or more

54. How many <u>non-prescription</u> medications do you currently receive from:

VA Pharmacy

- ☐ None
- ☐ 1 - 3
- ☐ 4 - 6
- ☐ 7 - 9
- ☐ 10 or more

Non-VA Pharmacy

- ☐ None
- ☐ 1 - 3
- ☐ 4 - 6
- ☐ 7 - 9
- ☐ 10 or more
